# Dermoscopy of Epidermization of Lip Indicating Focal Compact Sebaceous Metaplasia

**DOI:** 10.5826/dpc.1103a26

**Published:** 2021-07-08

**Authors:** Shailesh Malani, Bhushan Madke

**Affiliations:** 1 My Skin,Sailu, Dist, Parbhani, Maharashtra; 2 Department of Dermatology, Jawaharlal Nehhru Medical College, Datta Meghe Institute of Medical Sciences, Wardha, Maharashtra

**Keywords:** Dermoscopy, sebaceous metaplasia, epidermization of lip

## Introduction

Epidermization of the lip has been described as the presence of a smooth, steep, sharp leukokeratotic plaque over the lower vermilion border, blending evenly into the distal skin surface with an irregular proximal margin [1].

## Case Reports

**Figure 1:**
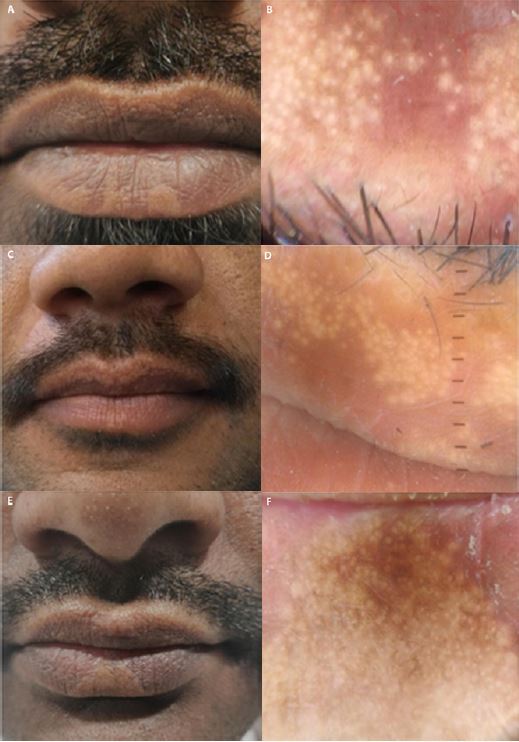
Clinical photos showing smooth, steep, sharp whitish plaques over lower as well as upper vermilion blending evenly into skin surface with irregular proximal margin. Dermoscopy of these lesions at 20 × magnifications showing multiple yellow dots with central opacity coalescing at the central part of the lesion and spaced wider at the periphery

We had 5 patients with similar clinical presentation. Among the examined patients, 2 of them presented both upper and lower vermilion region involvement. Dermoscopy of these lesions was performed using polarized contact dermoscopy (Dermlite DL4, 3Gen Inc. California, USA) Photographs were taken by One Plus 6T™ smart phone rear camera, equipped with an adaptor. We detected multiple yellow dots coalescing at the central part of the lesion and spacing wider at the periphery (Figure 1).

**Figure 2:**
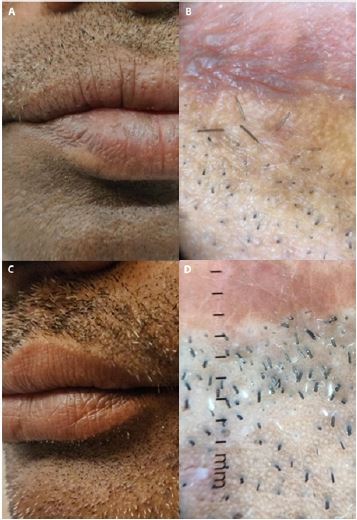
Dermoscopy showing additional features like terminal hairs in two cases and shiny white cone-shaped struts at follicular opening suggestive of the tail of in one case.

Few discrete dots were present at remote sites over the same or the other lip. Terminal hairs were found centrally, in a limited number of yellow dots. This was reported in 2 patients (Figure 2).

In 1 case, dermoscopy showed shiny white, cone-shaped struts at follicular openings suggestive of the tail of Demodex mite.

## Discussion

Literature suggests that histology of epidermization of the lip shows hyperkeratosis without parakeratosis or cellular atypia but dermoscopy findings in our patients suggest the presence of multiple ectopic sebaceous glands (Fordyce spots) coalescing to form a plaque/patch [1,2].

Differential diagnosis of epidermization of lips on dermoscopy includes leukoplakia and lip vitiligo. Dermoscopy of leukoplakia shows irregularly shaped white structureless areas of varying intensity along with whitish-pink clods and thick lines, while lip vitiligo presents a pearly white area of depigmentation with dotted blood vessels [2]. Dermoscopy can easily distinguish between these differences and epidermization can be treated with electro cauterization, ablation by CO_2_ laser, chemical cauterization by trichloroacetic acid or punch excision.

Yellow dots with central opaque areas have been described in case of Fordyce spots of the lip. Also, we found terminal hairs in 2 cases and Demodex mite in 1 case [2].

Epidermization is a condition in which tissues undergo epidermis-like changes including stratification and appendages’ presence, such as hairs, sebaceous glands, and eccrine glands. It is commonly found and described in the uterine cervix. Epidermization of the lip is not discussed in the literature as the vermilion part of the lip is covered with layer showing transition between epidermis and epithelium with partial stratification and not actual epidermis nor epithelium.

We suggest that Fordyce spots and epidermization of the lip are spectra of sebaceous metaplasia of labial epidermis with discrete involvement giving rise to Fordyce spots while focal compact involvement results in epidermization.

Metaplasia occurs when one differentiated cell type transforms in another differentiated cell type. This mechanism can be due to signaling cascade activation caused by cytokines’ activity, to growth factors, or to coordinated intracellular activity underlying stem cells’ reprogramming through various tissue-specific and differentiation genes.

Stem cells are known to exist in normal tissue and there is no change in phenotype of differentiated cell type. Metaplasia may be physiological or pathological and described either for epithelial or mesenchymal differentiation. Epithelial metaplasia is commonly seen at the transitional zones such as the junction at the level of the esophageal and gastric mucosa or the pharyngeal and bronchial mucosa.

Similarly, sebaceous metaplasia of the lip is an epidermal metaplasia where the pilosebaceous type of cells appear over the transitional zone of the labial epidermis. This is probably due to the hormonal stimulation deriving from the Hedgehog pathway activation (SHH) of stem cells located *in-situ*, resulting in sebaceous gland development. The male predominance and the onset at puberty might support the role of an androgen-dependent process.

For these reasons, smooth, steep, sharp whitish plaques’ formation over the vermilion cutaneous junction at the level of the lip, would be better defined as focal compact sebaceous metaplasia of the lip, rather than epidermization of the lip.
